# Supplementary material for: Alternate atxA and acpA dependent response of Bacillus anthracis to serum, HCO3- and CO2
Source: PLoS One. 2023 Feb 16;18(2):e0281879. doi: 10.1371/journal.pone.0281879 (PMC9934324; doi:10.1371/journal.pone.0281879)
Supplement: S1 Appendix — (DOCX) [file pone.0281879.s001.docx]

**Supplement 1**

To validate these findings, we used a previously reported VollumΔpXO2 chimera in which we substituted the genomic *bclA* gene (which is an exosporium glycoprotein non-essential for spore formation or virulence) with a CAP operon altered to be regulated by the PA_prom_ (**Table 1**). Thus, this mutant serves here as an indicator for AtxA dependent activation of the PA promotor, which results in capsule production (assayable readout). This mutant was then used as the basis for another mutant strain, in which the *atxA* gene was deleted, on top of the existing genetic alterations (**Table 1**). Thus, the exact role *atxA* plays in the regulation on the PA-promotor under the tested conditions could be resolved.

While 10% CO_2_ atmosphere induced capsule production in the Vollum wild type strain, little or no capsule production could be detected in the VollumΔpXO2Δ*bclA*::*pagA_prom_*-capA-E chimeric strain (**Figure S1**). Supplementing sDMEM with 0.75% HCO_3_^-^ resulted in capsule production in the Vollum and VollumΔpXO2Δ*bclA*::*pagA_prom_*-capA-E chimera. However, deletion of the *atxA* gene, in the background of the chimera mutant described, resulted in loss of capsule production (VollumΔpXO2Δ*bclA*::*pagA_prom_*-capA-EΔ*atxA*, **Figure S1**). Similar results were obtained by supplementing sDMEM with 10% NRS. These results complement the PA secretion experiment, indicating that HCO_3_^-^ and NRS induce toxin secretion in an *atxA* dependent manner.


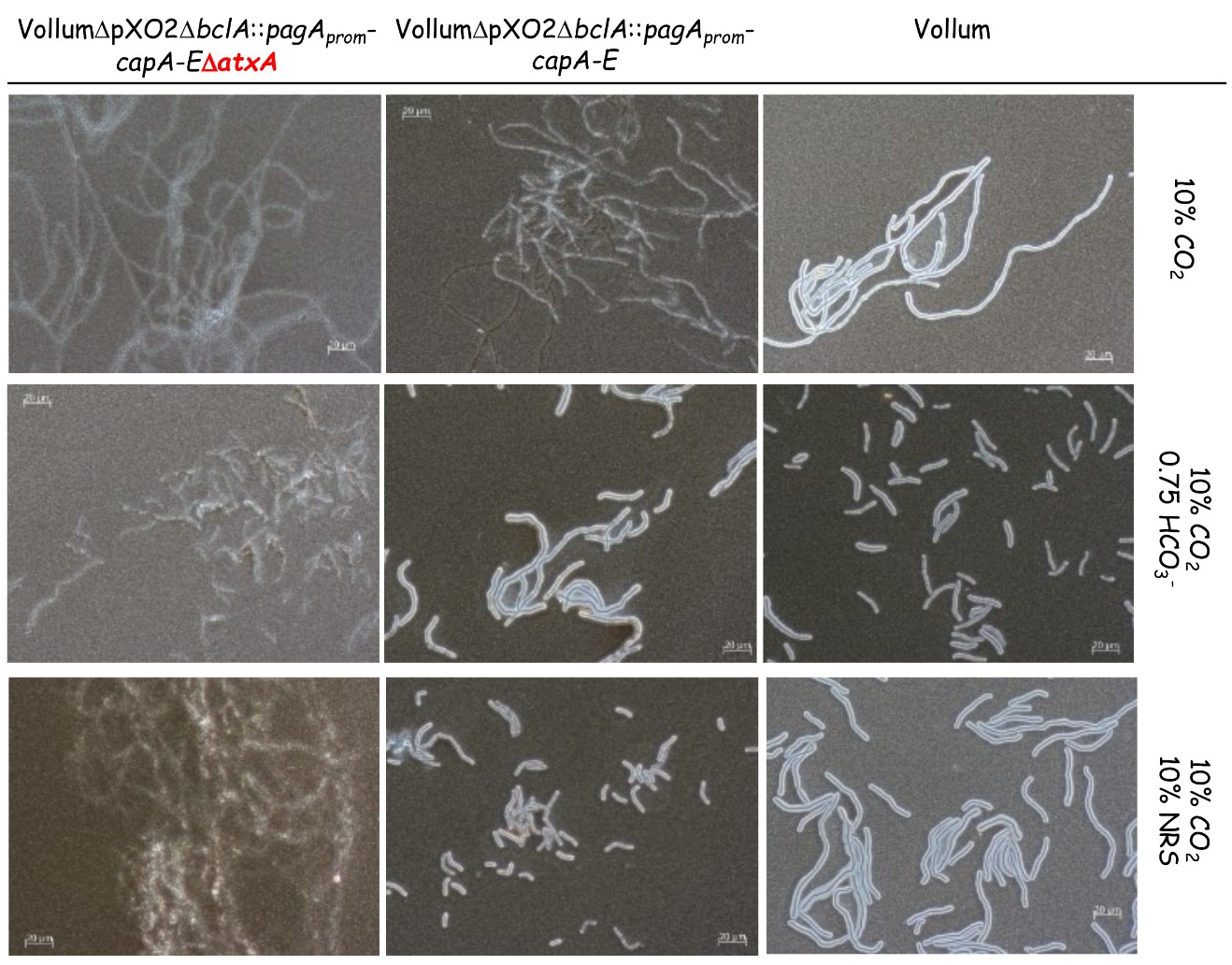


Figure S1. Capsule production in response to HCO_3_^-^ in a PA_prom_- regulated genomic CAP operon. Spores were seeded into 100μl of sDMEM as is or supplemented with 0.75% HCO_3_^-^ or 10% NRS and incubated at 37^o^C under an atmosphere of 10% CO_2_ for 24h. Capsule was imaged by India ink negative staining (capsule presence forms a typical bright outer layer).The different mutations are indicated (top panel).
